# Extinction Threat to a Previously Undescribed Species of Gall Wasp (Hymenoptera: Cynipidae) and Two Associated Parasitoid Species (Hymenoptera: Braconidae and Eulophidae) on a Threatened Rose

**DOI:** 10.1093/aesa/saad004

**Published:** 2023-03-27

**Authors:** Yoshihisa Abe, Tatsuya Ide, Kazunori Matsuo, Kaoru Maeto, Yajiao Wu

**Affiliations:** Faculty of Social and Cultural Studies, Kyushu University, 744 Motooka, Fukuoka 819-0395, Japan; Department of Zoology, National Museum of Nature and Science, 4-1-1 Amakubo, Tsukuba, Ibaraki 305-0005, Japan; Faculty of Social and Cultural Studies, Kyushu University, 744 Motooka, Fukuoka 819-0395, Japan; Graduate School of Agricultural Science, Kobe University, Kobe, Hyogo 657-8501, Japan; Graduate School of Integrated Sciences for Global Society, Kyushu University, 744 Motooka, Fukuoka 819-0395, Japan

**Keywords:** *Aprostocetus*, conservation, *Diplolepis*, *Rosa*, *Syntomernus*

## Abstract

*Diplolepis ogawai* Abe and Ide sp. nov. (Hymenoptera: Cynipidae) induces galls on *Rosa hirtula* (Regel) Nakai (Rosales: Rosaceae), which is endemic to a restricted area of Honshu, the main island of Japan. The gall is induced mainly on the leaf of *R*. *hirtula* in spring and the mature gall falls to the ground in early summer. The gall-inducing wasp emerges from the gall on the ground in the following spring, suggesting that *D*. *ogawai* is univoltine. From spring to summer, the braconid *Syntomernus flavus* Samartsev and Ku and the eulophid *Aprostocetus* sp. are parasitic on the larva of *D*. *ogawai* in the gall, and the adult wasp of both parasitoid species emerges from the gall on the ground in summer. For *S*. *flavus*, this is the first distribution record in Japan and the first host record. Since *R*. *hirtula* is threatened with extinction by succession and deforestation, *D*. *ogawai* and its two parasitoid wasp species are considered to be at risk of coextinction with the threatened rose. In the event that the population size of this rose species is further reduced, *D*. *ogawai* and its parasitoids may ­become extinct prior to the extinction of *R*. *hirtula*. To conserve these three wasp species associated with *R*. *hirtula*, protection of remnant vegetation where individuals of this threatened rose species grow is necessary.

To protect endangered and vulnerable species of wild fauna and flora, conservation of their habitats is important (Noss et al. 1997). A better understanding and increased interest among citizens regarding the biodiversity crisis are necessary in order to promote measures to conserve threatened species. For this reason, the Ministry of the Environment in Japan published the ‘Red Data Book 2014-Threatened Wildlife of Japan-’. Among the vulnerable plant species designated as ‘threatened class II’, *Rosa hirtula* (Regel) Nakai (Rosales: Rosaceae) is endemic to a limited area of Kanagawa, Shizuoka, and Yamanashi Prefectures on the main island of Honshu, Japan (Ministry of the Environment of Japan 2015). This rose species is found in thickets and their margins, and also on marshy slopes at 800–1,800 m above sea level (Ohba 2016). The decrease in the population size over the last 10 years and the probability of extinction within 100 years were estimated to be approximately 31% and 82%, respectively (Ministry of the Environment of Japan 2015). The main threats facing the continued survival of *R*. *hirtula* are succession and deforestation (Ministry of the Environment of Japan 2015). Host plant species sometimes harbor diverse insect communities on them (Strong et al. 1984), and in many cases herbivorous insects are quite host specific and hence reliant upon the host plant’s viability (Cardoso et al. 2011). If the host plant as a keystone species is at risk of extinction, then there is a risk of a cascade of extinctions of the associated herbivores and their natural enemies (Begon et al. 1996).

Rose gall wasps (Hymenoptera: Cynipidae: Diplolepidini), which consist of the genera *Diplolepis* Geoffroy and *Liebelia* Kieffer, are gall inducers associated with *Rosa* spp. (Csóka et al. 2005, Ronquist et al. 2015). The global phylogenetic relationships among members of the genus *Diplolepis* have been inferred, suggesting that this genus is composed of a Holarctic leaf-galler clade and a Nearctic stem-galler clade (Zhang et al. 2019, 2020). However, the biogeographic origin of this genus has not yet been determined, mainly due to the lack of information about eastern Palearctic species (Zhang et al. 2020). Based on the substantial flora of *Rosa* spp. in China (Ohba 1997), the existence of a potentially diverse fauna of rose gall wasps in the eastern Palearctic was pointed out (Abe et al. 2007). In recent years, taxonomic studies on *Diplolepis* in this region were conducted and several new species were discovered (Wang et al. 2013, Pujade-Villar et al. 2020, Zhu et al. 2021).

During a field survey of insect galls, Haruo Ogawa discovered galls on *R*. *hirtula* shrubs. He observed that gall wasps and their parasitoids emerged from the galls he reared. Preliminarily, morphological examinations of these wasps revealed that the gall wasp was a member of *Diplolepis*, and the parasitoids consisted of one braconid and one eulophid species. Many rare and undescribed species of insects become extinct before description (Pimm et al. 2014). In terms of biodiversity conservation, it is important to give a scientific name to a threatened and undescribed species to facilitate communication about it among scientists (Braby and Williams 2016). Because the galls induced by an undescribed species of *Diplolepis* are only found in a few places, describing the new gall wasp species and identifying both parasitoid species are urgently required. In the present study, in addition to revealing a previously unknown insect fauna on the vulnerable plant, formal description or identification of that Hymenopteran fauna and partial sequences of the mtDNA COI region of these three species are provided. Furthermore, the conservation of the wasps associated with the threatened rose species is discussed.

## Materials and Methods

### Collection, Rearing, and Morphological Examination

In 2020 and 2021, fresh galls that had fallen to the ground from *R*. *hirtula* shrubs were collected from a few localities in Kanagawa and Shizuoka Prefectures, Japan, by Haruo Ogawa (Table 1). After keeping these galls in shade under field conditions in Susono City, Shizuoka Prefecture, the adults of the gall wasps and their parasitoids emerged from the galls. The wasps were collected and stored in 99% ethanol for morphological observation and DNA analysis. Some specimens of the new species of rose gall wasp, *Diplolepis ogawai*, and its parasitoids were dried and examined under stereomicroscopes (models S8APO and MZ APO, Leica Microsystems KK, Tokyo, Japan) fitted with digital single-lens reflex camera (model E-30, Olympus, Tokyo, Japan), and under a scanning electron microscope (model JSM-5600LV, JEOL, Tokyo, Japan) at 1.5 kV. The length of body parts was measured with an ocular micrometer. Focus stacking was performed using CombineZP software (available from: https://combinezp.software.informer.com/) for the microscopic images. All images were processed and assembled using the GNU Image Manipulation Program (GIMP 2.10.20; available from https://www.gimp.org/).

**Table 1.** Specimens of *Diplolepis ogawai*, *Syntomernus flavus*, and *Aprostocetus* sp.

Species	Individuals	Locality	Collecting	Adult emergence	Depository	Note	GenBank accession number
*Diplolepis ogawai*	1 female	Hakone, Kanagawa Pref.	7-23. VI. 2020	10. IV. 2021	BLKU	Holotype	–
	1 female	Hakone, Kanagawa Pref.	7-23. VI. 2020	10. IV. 2021	BLKU	Paratype	–
	1 female	Hakone, Kanagawa Pref.	7-23. VI. 2020	11. IV. 2021	NSMT	Paratype	OP281689
	1 female	Hakone, Kanagawa Pref.	7-23. VI. 2020	11. IV. 2021	NSMT	Paratype	–
*Syntomernus flavus*	1 female	Hakone, Kanagawa Pref.	21. VI. 2020	24. VI. 2020	BLKU	–	OP281690
	1 female	Fujinomiya City, Shizuoka Pref.	24. VI. 2021	11. VII. 2021	NSMT	–	–
	1 female and 4 males	Fuji City, Shizuoka Pref.	15. VII. 2021	27-29. VII. 2021	BLKU, NSMT	–	–
*Aprostocetus* sp.	8 females and 1 male	Fuji City, Shizuoka Pref.	15. VII. 2021	6. VIII. 2021	BLKU	–	–
	1 female	Hakone, Kanagawa Pref.	18. VII. 2021	25. VII. 2021	–	–	OP281691
	2 females and 3 males	Hakone, Kanagawa Pref.	18. VII. 2021	25. VII. 2021	BLKU	–	–
	1 female	Fuji City, Shizuoka Pref.	18. VII. 2021	2. VIII. 2021	–	–	OP281692
	5 females and 3 males	Fuji City, Shizuoka Pref.	18. VII. 2021	2. VIII. 2021	BLKU	–	–

BLKU: Biosystematics Laboratory, Faculty of Social and Cultural Studies, Kyushu University.

NSMT: Department of Zoology, National Museum of Nature and Science.

In the description of *D*. *ogawai*, the following morphological abbreviations are used: POL, postocellar line (the distance between the inner edges of the two lateral ocelli); OOL, ocular–ocellar line (the distance from the outer edge of the lateral ocellus to the compound eye); and LOL, lateral–ocellar line (the distance between the median and lateral ocelli). The morphological terminology for gall wasps follows Ronquist and Nordlander (1989), Melika (2006), and Liljeblad et al. (2008); the description of surface sculptures follows Harris (1979).

### Examination of Galls

The shape of nine galls from which wasps of *D*. *ogawai* and its parasitoids emerged was observed and their diameters were measured with digital calipers to clarify the effects of parasitism on gall shape and size. The galls were then dissected under a binocular stereomicroscope to determine their contents. To compare the structure of the galls on *R*. *hirtula* by *D*. *ogawai* with that of the galls induced by *Diplolepis japonica* (Walker), galls of the latter species were collected from leaves of *Rosa multiflora* Thunb. by Yoriko Abe in Kyonan-cho, Musashino City, Tokyo on 25 May 2021 and were reared under field conditions on the Ito Campus of Kyushu University until 26 March 2022 when the female wasps emerged. These wasps were identified as *D*. *japonica* based on Yasumatsu and Taketani (1967).

### DNA Analysis

Partial sequences (658 bp) of the COI gene of mtDNA were determined for one wasp of *D*. *ogawai* and that of the braconid using previously described methods (Ide and Abe 2019). DNA of two eulophid specimens were destructively extracted after morphological identification. The primers used in the analysis of the eulophid were: forward; COI_pF2 5ʹ-ACC WGT AAT RAT AGG DGG DTT TGG DAA-3ʹ and reverse; COI_2437d 5ʹ-GCT ART CAT CTA AAW AYT TTA ATW CCW G-3ʹ (Kaartinen et al. 2010). PCR conditions were as follows: 94°C for 2 min, followed by four repeated cycles of 94°C for 30 s, 45°C for 1 min, and 72°C for 1 min. These steps were followed by 34 repeated cycles of 94°C for 30 s, 50°C for 1 min, and 72°C for 1 min, before storage at 4°C. The determined sequences were deposited in GenBank (https://www.ncbi.nlm.nih.gov/genbank/) under the accession numbers OP281689 for *D*. *ogawai*, OP281690 for the braconid and OP281691, OP281692 for the eulophid. The COI sequence of *D*. *ogawai* was compared with those of other congeners in GenBank using BLAST (https://blast.ncbi.nlm.nih.gov/Blast.cgi; accessed 31 January 2022).

### Depository of Voucher Specimens

The data of all the sample details are provided in Table 1.

### Nomenclature

This paper and the nomenclatural act(s) it contains have been registered in Zoobank (www.zoobank.org), the official register of the International Commission on Zoological Nomenclature. The LSID (Life Science Identifier) number of the publication is: urn:lsid:zoobank.org:pub:89AAB7FF-3A3B-49B1-9493-D67A0FB72B4A.

## Results

### Species Identification

Morphological differences between *D*. *ogawai* and other congeners were detected and this new species is described below. The parasitoids reared from the new *Diplolepis* were identified as *Syntomernus flavus* Samartsev and Ku (Hymenoptera: Braconidae) and *Aprostocetus* sp. (Hymenoptera: Eulophidae) based on Samartsev and Ku (2020) and Graham (1987), respectively.

### Taxonomy

#### *Diplolepis ogawai* Abe and Ide, sp. nov.

(urn:lsid:zoobank.org:act:24F3E6F2-081B-4A3A-A518-FDF2157E7785)

[Japanese name: Sanshôbara-ha-tamabachi], which means *Rosa hirtula*-leaf-gall wasp in Japanese

(Figs. 1 and 2)

**Fig. 1.**
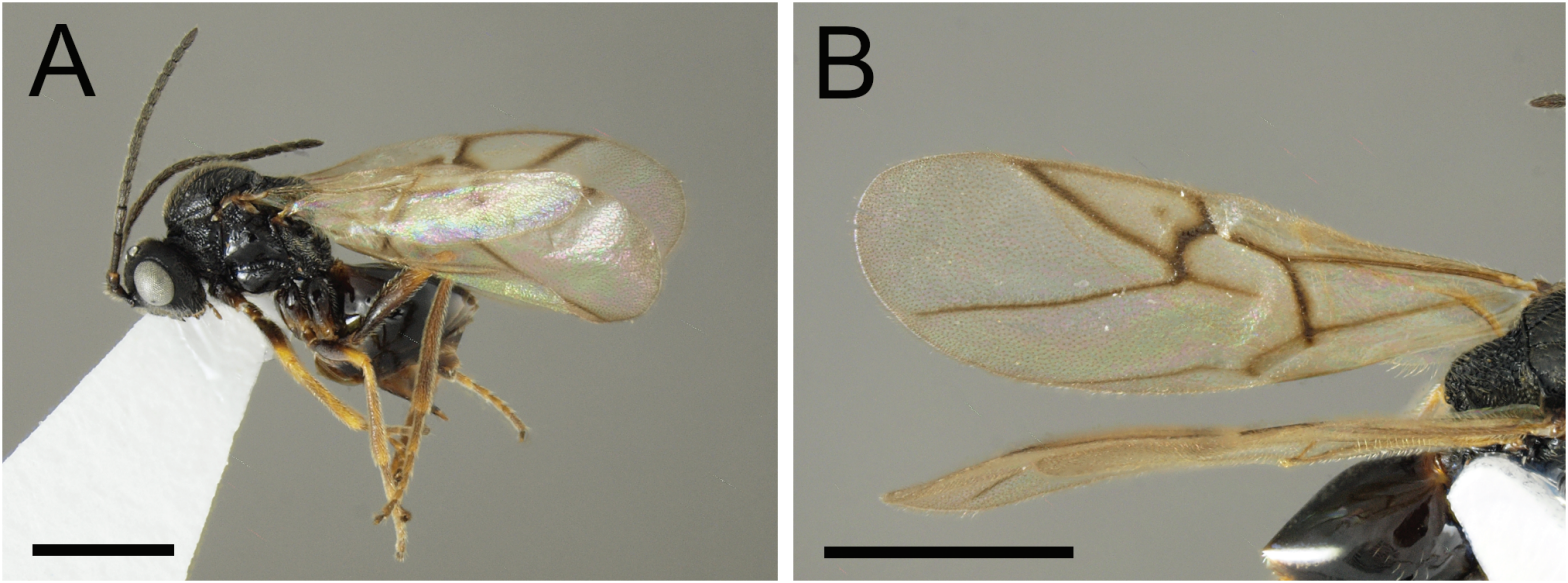
*Diplolepis ogawai* (scale bar = 1 mm). (A) Habitus (holotype). (B) Forewing (paratype).

**Fig. 2.**
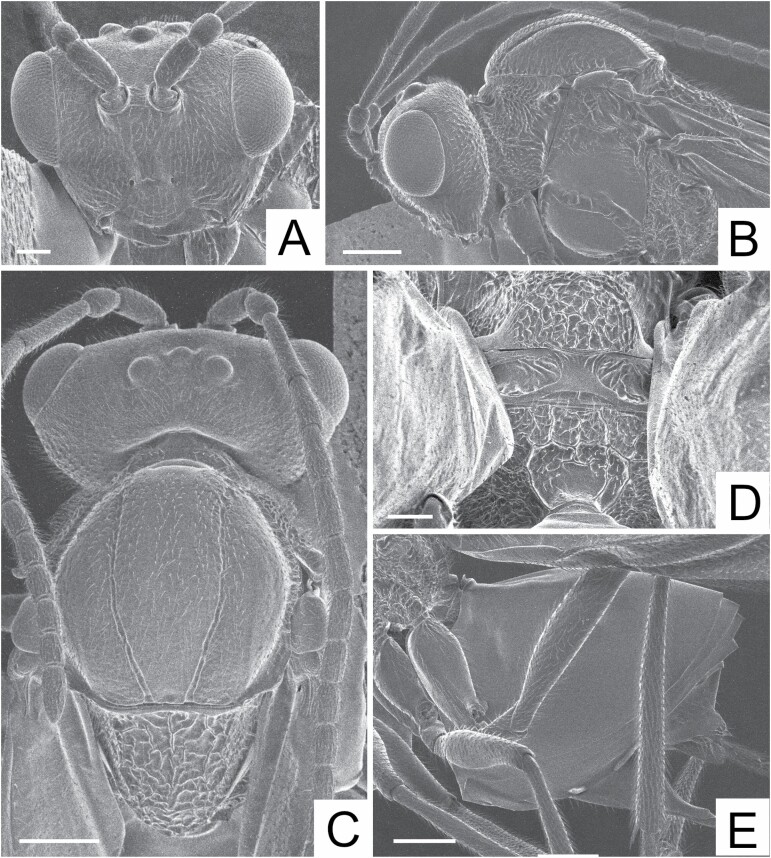
*Diplolepis ogawai* (holotype). (A) Head, anterior view (scale bar = 0.1 mm). (B) Head and mesosoma, lateral view (scale bar = 0.2 mm). (C) Head and mesosoma, dorsal view (scale bar = 0.2 mm). (D) Mesosoma, postero-dorsal view (propodeum) (scale bar = 0.1 mm). (E) Metasoma, lateral view (scale bar = 0.2 mm).

#### Specimens Examined

See Table 1.

#### Female Holotype

Body (Fig. 1A) black. Mandible yellowish brown with brown tip. Maxillary and labial palpi, tegula, hypopygial spine brown. Wings hyaline; wing veins brown. Legs partially yellowish brown.

Head (Figs. 2A and C) setose, coriarious, slightly broader than mesosoma in dorsal view. Ocelli round; POL:OOL:LOL = 27:31:12. Distance between antennal rims 0.4 times as great as distance between lateral margin of antennal rim and inner margin of compound eye. Inner margins of compound eye almost parallel. Ventral margin of clypeus rounded. Gena not broadened behind eye in frontal view. Antenna 14-segmented; scape except for base 1.9 times as long as pedicel; relative lengths of flagellomeres 1–12: 47, 25, 25, 24, 22, 22, 20, 19, 17, 17, 17, 28.

Mesosoma (Figs. 2B–D) dorsally convex, longer than height in lateral view, setose except for mesopleuron. Pronotum dorsomedially narrow, rugose laterally. Mesoscutum coriarious; notaulus percurrent; anteroadmedian and parapsidal signa (anterior parallel and parapsidal lines) obscurely present; median mesoscutal impression (median mesoscutal line) absent. Mesoscutellum rugose; scutellar fovea absent. Mesopleuron smooth, shining with broad crenulate furrow; mesopleural triangle rugose; ventral border of mesopleural triangle not clearly marked by ledge. Metascutellum subrectangular; metanotal trough smooth with some wrinkles. Propodeum anteriorly and laterally rugose; lateral propodeal carinae anteriorly with three longitudinal carinae, curved outward in posterior 2/3, delimiting a closed area.

Wing surface and margin closely ciliated. Marginal cell of forewing closed with pale anterior margin, 2.3 times as long as wide, 2r curved without median prolongation into the marginal cell. Areolet indistinct. Hind femur without flange. Apex of metatarsal claw bent; base not expanded to lobe. Lengths of forewing and hind tibia 2.75 and 0.96 mm, respectively.

Metasoma (Fig. 2E) smooth, longer than mesosoma in lateral view. Metasomal tergum II large (more than half of metasoma) with sparse setae laterally.

#### Male

Not known.

#### Variation

Areolet indistinct or absent. Lengths of forewing (*n* = 4) 2.75–3.11 mm, and of hind tibia 0.96–1.03 mm.

#### Remarks

The genus *Diplolepis* contains 46 species worldwide (Zhu et al. 2021). Of these, *D*. *japonica* has been recorded in Japan (Yasumatsu and Taketani 1967). This species is also native to Korea and China (Wang et al. 2013, Ide and Abe 2020), but the Chinese population could be an undescribed species (Pujade-Villar et al. 2020). The *Diplolepis ogawai* female can be distinguished from the *D*. *japonica* female by the following four morphological characteristics: 1) metasoma is dark brown and partially reddish-brown in *D*. *japonica*, but black in *D*. *ogawai*; 2) mesoscutal impression is present in *D*. *japonica*, but absent in *D*. *ogawai*; 3) areolet is distinct in *D*. *japonica*, but indistinct or absent in *D*. *ogawai*; 4) forewing is infuscate along all the veins of the marginal cell in *D*. *japonica*, but not infuscate in *D*. *ogawai* (Fig. 1B).

*D. ogawai* is most similar to the Chinese species, *Diplolepis valtonyci* Zhu, Wang, and Pujade-Villar. The *D*. *ogawai* female can be distinguished from the *D*. *valtonyci* female by the following three morphological characteristics: 1) metasoma is chestnut brown in *D*. *valtonyci*, but black in *D*. *ogawai*; 2) mesoscutal impression is present in *D*. *valtonyci*, but absent in *D*. *ogawai*; 3) forewing is infuscate along all the veins of the marginal cell in *D*. *valtonyci*, but not infuscate in *D*. *ogawai*.

#### Etymology

The new species is named in honor of Haruo Ogawa, who collected the specimens.

#### Gall

**Fig. 3.**
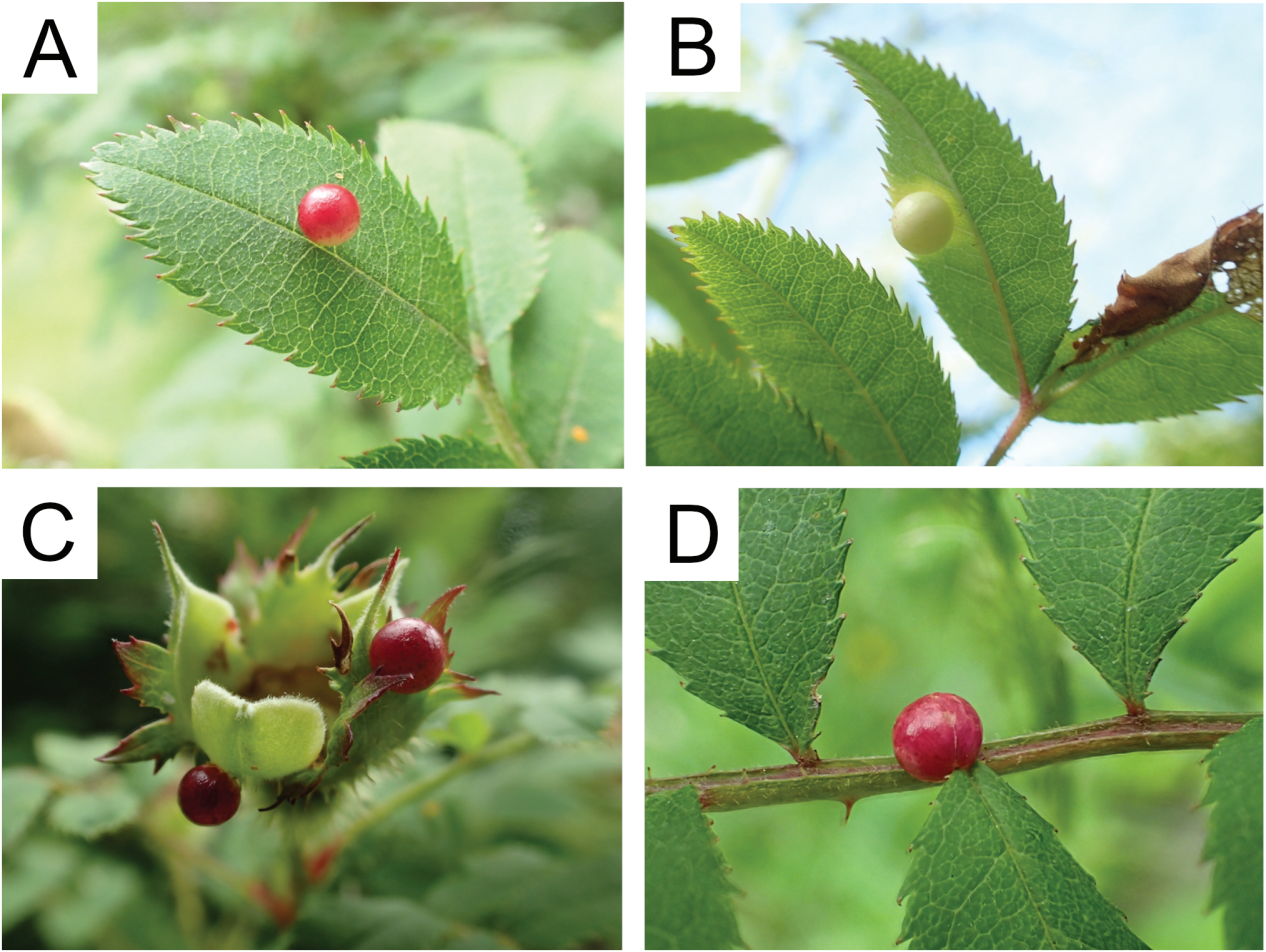
Galls of *Diplolepis ogawai* (photo by Haruo Ogawa). (A) A gall on the upper surface of a *Rosa hirtula* leaf. (B) A gall on the lower surface of a *Rosa hirtula* leaf. (C) Two galls on sepals of *Rosa hirtula*. (D) A gall on a petiolule of *Rosa hirtula*.

According to personal observations of Haruo Ogawa, the galls of *D*. *ogawai* can be characterized as follows. Galls are induced on both the upper and lower surfaces of the *R*. *hirtula* leaf (Figs. 3A and B), and also on the sepal (Fig. 3C) and petiolule (Fig. 3D). They are spherical and smooth without spines. Galls are white, pink, or red when on the host plant, but turn brown after falling on the ground.

**Fig. 4.**
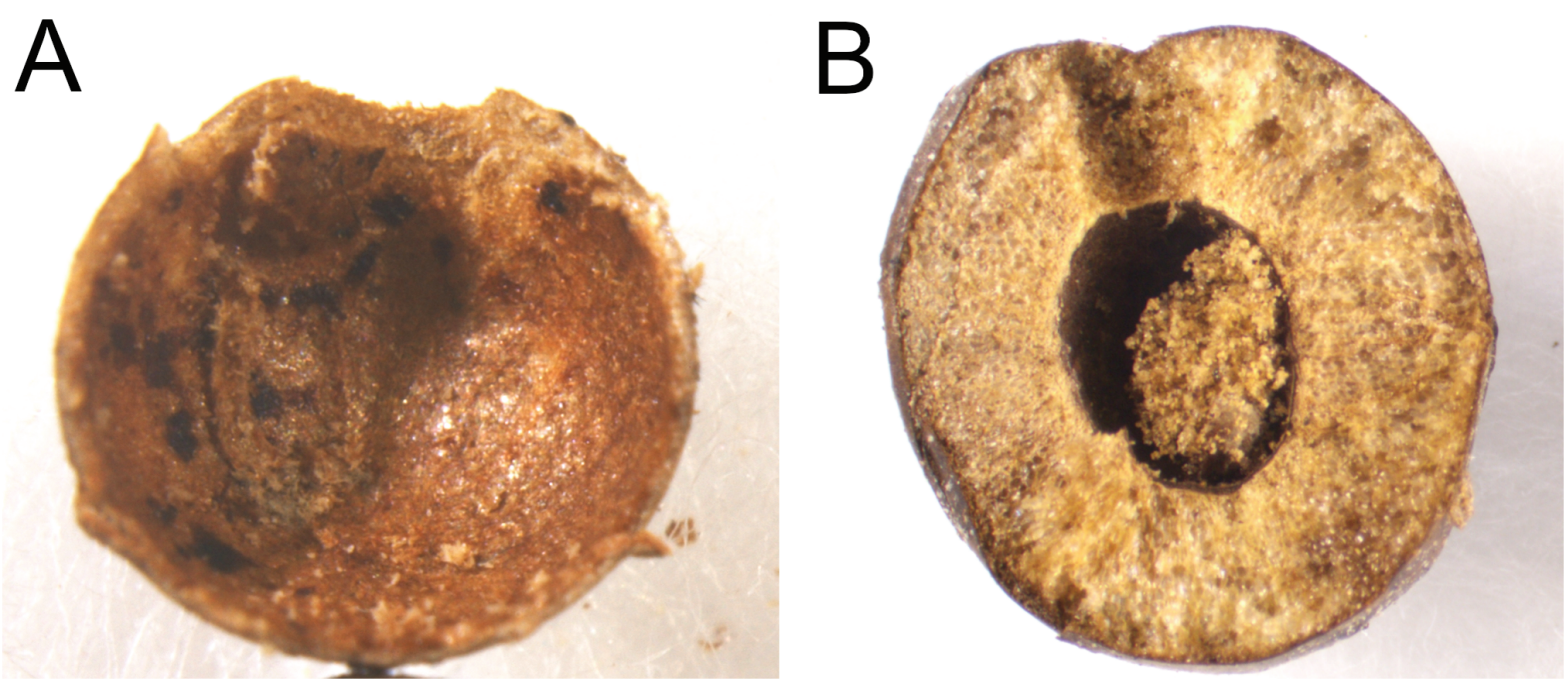
Longitudinal section of *Diplolepis ogawai* and *D*. *japonica* galls from which adult gall wasps emerged. (A) A gall of *Diplolepis ogawai*. (B) A gall of *Diplolepis japonica*.

Each gall has one larval chamber (Fig. 4A). The diameter of the galls from which the gall-inducing wasps emerged was 3.3–3.4 mm (*n* = 4). In contrast, the gall induced by *D*. *japonica* bears sharp-pointed spines (Yasumatsu and Taketani 1967) and the maximum diameter of galls from which *D*. *japonica* females emerged was 6.8–9.8 mm (*n* = 5). The gall wall is thin (ca. 0.2 mm) in *D*. *ogawai*, but thick (2.0–2.7 mm) in *D*. *japonica* (Fig. 4). According to Yasumatsu and Taketani (1967), the diameter and wall of *D. japonica* gall are typically 8–9 mm and 1.5–2.0 mm, respectively.

#### Host Plant

*Rosa hirtula* (Regel) Nakai (family: Rosaceae)

#### Geographic Distribution

Japan (Kanagawa and Shizuoka Prefectures in Honshu).

#### Life Cycle

A univoltine life cycle was indicated by rearing the wasps (Table 1).

#### *Syntomernus flavus* Samartsev and Ku, 2020

(Fig. 5A)

**Fig. 5.**
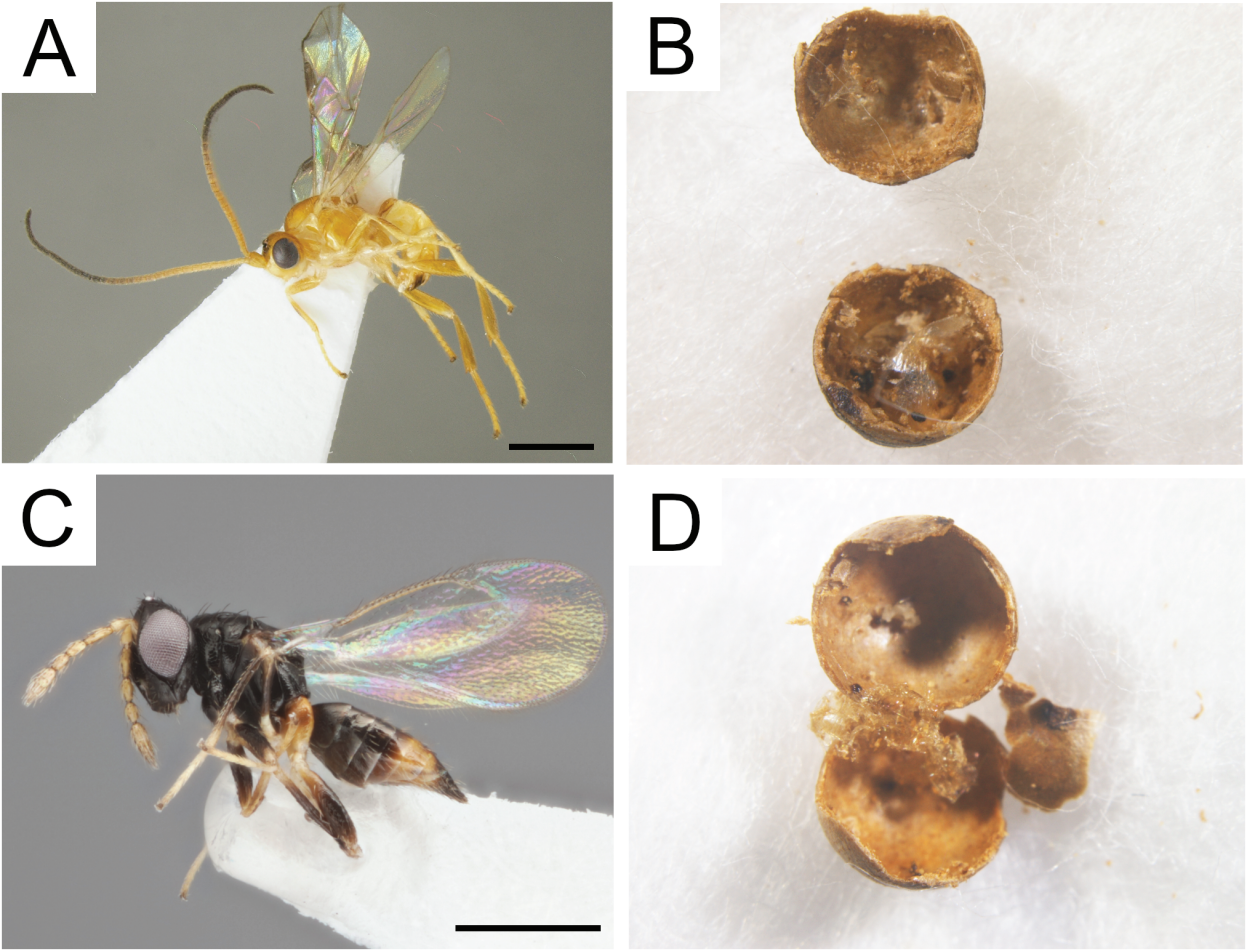
Parasitoids and longitudinal section of *Diplolepis ogawai* galls from which adult parasitoid wasps emerged. (A) Habitus of a male of *Syntomernus flavus* Samartsev and Ku (scale bar = 1 mm). (B) A longitudinal section of gall from which an adult wasp of *Syntomernus flavus* emerged. (C) Habitus of a female of *Aprostocetus* sp. (scale bar = 0.5 mm). (D) A longitudinal section of gall from which adult wasps of *Aprostocetus* sp. emerged.

#### Specimens Examined

See Table 1.

#### Host

*Diplolepis ogawai* Abe and Ide

#### Geographic Distribution

South Korea (Samartsev and Ku 2020), Japan (Kanagawa and Shizuoka Prefectures in Honshu). New to Japan.

#### Remarks

*S. flavus* was previously only known from Korea and its host was unknown (Samartsev and Ku 2020). For *S*. *flavus*, Japan is a new distributional record. Japanese specimens were identical to the original description of the South Korean specimens (Samartsev and Ku 2020), except that the pterostigma was entirely brown (without a basal yellow patch) in some males (Fig. 5A).

Integumentary fragments of the gall wasp larva were found in two galls from which *S*. *flavus* wasps emerged (Fig. 5B). Because only a single braconid wasp emerged from any particular host gall and no dead specimens of *S*. *flavus* were found inside, this braconid species is considered to be a solitary parasitoid. The diameters of the two host galls were 3.4 and 3.5 mm. No difference in gall shape and size was observed between galls fostering *S*. *flavus* and those fostering *D*. *ogawai*.

The members of *Syntomernus* include parasitoids of both cecidomiid gall midges and insect larvae inside dipterocarp fruits; some species of this genus have been reared from fig syconia and one species is known to be phytophagous on syconium tissues (Samartsev and Ku 2020 and references therein). Based on the findings of this study, *S*. *flavus* is considered to be a solitary parasitoid of *D*. *ogawai* larva. This is the first record of the genus *Syntomernus* associated with a cynipid gall inducer. This braconid parasitoid depends on *D*. *ogawai* from spring to summer. Because the galls of *D*. *ogawai* fall to the ground in early summer, no fresh galls induced by *D*. *ogawai* are present on *R*. *hirtula* when the adult wasp of *S*. *flavus* emerges from its host gall on the ground in summer. Therefore, the host(s) of *S*. *flavus* from summer to the following spring is unknown.

#### *Aprostocetus* sp.

(Fig. 5C)

#### Specimens Examined

See Table 1.

#### Host

*Diplolepis ogawai* Abe and Ide.

#### Geographic Distribution

Japan (Kanagawa and Shizuoka Prefectures in Honshu).

#### Remarks

The genus *Aprostocetus* contains more than 800 species worldwide (Noyes 2019). Of these, six species have been recorded in Japan (Matsuo 2020). Detailed morphological comparisons among the Japanese species are required for precise identification of the *Aprostocetus* sp. that was obtained in this study. However, due to the lack of detailed morphological descriptions and illustrations, substantial taxonomic efforts will be required in order to characterize the known Japanese species. For example, morphological information of *A. pallidipes* (Ashmead), a Japanese species, has not been amended since its original description (Ashmead 1904). However, since the taxonomic revision of the Japanese *Aprostocetus* is beyond the scope of this paper, the specimens obtained in this study have not been identified to the species level at present.

Integumentary fragments of wasp larvae were found in three galls from which *Aprostocetus* sp. wasps emerged (Fig. 5D). It is not clear whether they are from a cynipid larva or from some other hymenopteran larva, e.g., *S*. *flavus*. Because 6–9 wasps emerged from one host gall and no dead specimens of this eulophid were found in the galls, this eulophid species is considered to be a gregarious parasitoid. The diameters of the three host galls were 2.9, 3.5, and 3.5 mm. One gall was somewhat smaller than the others, but no difference in the shape and size was observed between galls fostering *Aprostocetus* sp. and those fostering *D*. *ogawai*.

As in *S*. *flavus*, the host(s) of *Aprostocetus* sp. from summer to the following spring is unknown.

### DNA Analysis

The BLAST analysis identified *Diplolepis radicum* (Osten Sacken), *Diplolepis rosae* (L.), and *Diplolepis fusiformans* (Ashmead) as having the most similar COI sequences to *D*. *ogawai*, with COI sequence homologies in the range 89–90% compared to *D*. *ogawai*.

In addition, the BLAST analysis selected *Baryscapus pallidae* Graham and *Aprostocetus cerricola* (Erdös) as the two species with COI sequences most similar to *Aprostocetus* sp. These two species had COI sequence homologies in the range 90–92% compared to *Aprostocetus* sp.

## Discussion

In this study, we described the gall wasp, *D*. *ogawai*. In addition, two parasitoids, *S. flavus* and *Aprostocetus* sp., were reared from *D*. *ogawai*. This is a new distributional record for *S. flavus* from Japan.

Both BLAST searches for *D*. *ogawai* and its parasitoid *Aprostocetus* sp. result in about 90% match. Moreover, the three best hits for *D*. *ogawai* are two Nearctic and one European species, and the two best hits for *Aprostocetus* sp. are European species. Such a situation is ascribable to the lack of appropriate sequence data from most of the Asian representatives of *Diplolepis* spp. and their parasitoids. Effective DNA barcoding requires a well-sampled, extensive reference database. Reliable DNA-based determination of phylogenetic position of this new species needs nuclear data as well as better Asian sampling.

Because the members of *Diplolepis* induce galls on plants of the genus *Rosa* (e.g., Zhang et al. 2020), the distributions of these gall wasps may be associated with those of specific species of *Rosa* (Sardón-Gutiérrez et al. 2021). However, with the exception of *Diplolepis spinosissimae* (Giraud) in *Rosa pimpinellifolia* L., no reliable data indicates that *Diplolepis* species are host-specific in Europe at the level of host plant species (Stille 1984, Kohnen et al. 2011). In the eastern Palearctic, *D*. *japonica* has been recorded from *R*. *multiflora* and *R*. *rugosa* (Yasumatsu and Taketani 1967, Wang et al. 2013). Since the distribution ranges of both plant species include Japan, Korea, China, and Russia (Nagamitsu 2017, Jeon and Kim 2019), *D*. *japonica* is widely distributed in East Asia, including Japan, Korea, and China (Yasumatsu and Taketani 1967, Wang et al. 2013, Ide and Abe 2020). However, according to Pujade-Villar et al. (2020), the Chinese population of *D*. *japonica* could be an undescribed species. In contrast to the typical *Diplolepis* species, galls of *D*. *ogawai* have been only recorded from *R*. *hirtula*. The exceptionally high host specificity of *D*. *ogawai* to *R*. *hirtula*, which has a very limited natural distribution, most likely means that the distribution range of this gall wasp is restricted to an area on the main island of Japan. The vulnerable rose *R*. *hirtula* is considered to be a keystone species within a local ecological community (a network in which species are trophically connected; e.g., Paine 1980), because the previously unknown biodiversity including three hymenopteran species depends on it. As suggested by Dunn et al. (2009), coextinction through cascading effects across trophic levels may be the most common form of biodiversity loss. Because the loss of a keystone species is likely to result in extinction of interdependent species (Koh et al. 2004a), conservation efforts should extend from *R*. *hirtula* to the newly discovered fauna associated with it.

Although some species of cynipid gall inducers often cause serious damage to their host plants (Stone et al. 2002), the rarity of *D*. *ogawai* means that it likely does not adversely affect the host plant population. Therefore, when *R. hirtula* suffers anthropogenic habitat degradation, the extinction of *D*. *ogawai* due to the decline of the host plant populations is rather likely to come earlier. *R. hirtula* is distributed in a limited area (Ohba 2016) and is threatened with extinction by a decrease in favorable habitats due to the progress of succession (Ministry of the Environment, Japan 2015). Moreover, loss of rose habitats due to deforestation is another concern (Ministry of the Environment of Japan 2015). In the present study, the previously unknown insect fauna depending on *R*. *hirtula* was discovered. The new gall wasp *D. ogawai* appears to be host-limited to the rare rose, and two parasitoids: *S*. *flavus* and *Aprostocetus* sp. depend on this gall wasp from spring to summer. These three wasp species are also considered to be at risk of coextinction with this vulnerable plant as follows: the primary extinction of *R*. *hirtula* causes the secondary extinction of *D*. *ogawai*, followed by the tertiary extinction of *S*. *flavus* and *Aprostocetus* sp. Generally, host-limited herbivorous insects may become extinct before their host plants (Koh et al. 2004b, Moir et al. 2010). If the population size of *R*. *hirtula* is reduced further, these three wasp species may become extinct before the vulnerable host plant. Therefore, the insect fauna depending on the rare rose is considered vulnerable. As pointed out by Cardoso et al. (2011) and Moir and Brennan (2020), criteria for listing threatened species should be modified to consider coextinction of species that depend on threatened hosts/prey. To conserve the new gall wasp species and its two parasitoid species, protection of areas with remnant populations of this vulnerable rose species is necessary.

## Notes

Version of Record, first published online March 27, 2023 with fixed content and layout in compliance with Art. 8.1.3.2 ICZN.
